# A chemical screen identifies structurally diverse metal chelators with activity against the fungal pathogen *Candida albicans*

**DOI:** 10.1128/spectrum.04095-23

**Published:** 2024-02-20

**Authors:** Sara Fallah, Dustin Duncan, Kyle D. Reichl, Michael J. Smith, Wenyu Wang, John A. Porco, Lauren E. Brown, Luke Whitesell, Nicole Robbins, Leah E. Cowen

**Affiliations:** 1 Department of Molecular Genetics, University of Toronto, Toronto, Ontario, Canada; 2 Department of Chemistry, Brock University, St. Catharines, Ontario, Canada; 3 Department of Chemistry, Center for Molecular Discovery (BU-CMD), Boston University, Boston, Massachusetts, USA; Stony Brook University, Stony Brook, New York, USA

**Keywords:** metal chelator, fungal pathogen, *Candida*, *Aspergillus*, iron, chemical library

## Abstract

*Candida albicans*, one of the most prevalent human fungal pathogens, causes diverse diseases extending from superficial infections to deadly systemic mycoses. Currently, only three major classes of antifungal drugs are available to treat systemic infections: azoles, polyenes, and echinocandins. Alarmingly, the efficacy of these antifungals against *C. albicans* is hindered both by basal tolerance toward the drugs and the development of resistance mechanisms such as alterations of the drug’s target, modulation of stress responses, and overexpression of efflux pumps. Thus, the need to identify novel antifungal strategies is dire. To address this challenge, we screened 3,049 structurally-diverse compounds from the Boston University Center for Molecular Discovery (BU-CMD) chemical library against a *C. albicans* clinical isolate and identified 17 molecules that inhibited *C. albicans* growth by >80% relative to controls. Among the most potent compounds were CMLD013360, CMLD012661, and CMLD012693, molecules representing two distinct chemical scaffolds, including 3-hydroxyquinolinones and a xanthone natural product. Based on structural insights, CMLD013360, CMLD012661, and CMLD012693 were hypothesized to exert antifungal activity through metal chelation. Follow-up investigations revealed all three compounds exerted antifungal activity against non-albicans *Candida*, including *Candida auris* and *Candida glabrata*, with the xanthone natural product CMLD013360 also displaying activity against the pathogenic mould *Aspergillus fumigatus*. Media supplementation with metallonutrients, namely ferric or ferrous iron, rescued *C. albicans* growth, confirming these compounds act as metal chelators. Thus, this work identifies and characterizes two chemical scaffolds that chelate iron to inhibit the growth of the clinically relevant fungal pathogen *C. albicans*

**IMPORTANCE:**

The worldwide incidence of invasive fungal infections is increasing at an alarming rate. Systemic candidiasis caused by the opportunistic pathogen *Candida albicans* is the most common cause of life-threatening fungal infection. However, due to the limited number of antifungal drug classes available and the rise of antifungal resistance, an urgent need exists for the identification of novel treatments. By screening a compound collection from the Boston University Center for Molecular Discovery (BU-CMD), we identified three compounds representing two distinct chemical scaffolds that displayed activity against *C. albicans*. Follow-up analyses confirmed these molecules were also active against other pathogenic fungal species including *Candida auris* and *Aspergillus fumigatus*. Finally, we determined that these compounds inhibit the growth of *C. albicans* in culture through iron chelation. Overall, this observation describes two novel chemical scaffolds with antifungal activity against diverse fungal pathogens.

## OBSERVATION

Although largely overlooked, fungal pathogens are major contributors to infectious disease, killing approximately 1.5 million people annually (1). Existing primarily as commensal members of the human mycobiota, *Candida* species can cause both superficial and life-threatening systemic infections in immunocompromised individuals (1). *Candida albicans* kills more than 400,000 individuals worldwide each year and is the fourth leading cause of nosocomial infections in the United States (1). Alarmingly, effective treatment of these infections is challenging due to the limited number of fungal-specific cellular targets, the increase in resistant clinical isolates, and a dearth of antifungal drug classes for the treatment of systemic infections.

The discovery of new antifungals remains a significant challenge. The Boston University Center for Molecular Discovery (BU-CMD) collection encompasses diverse chemical scaffolds of academic origin, curated in the absence of strict “drug-likeness” cutoffs (e.g., Lipinski’s rule of five or “Ro5”) and with a relaxed allowance for structural motifs known or suspected to cause pan-assay interference (e.g., *“*PAINS” compounds) (2–5). As a result, the collection represents an alternative to heavily curated Ro5 libraries and carries the potential for the discovery of new antimicrobials, which often exist outside of Ro5 chemical space (6). Our previous screen of a subset of the BU-CMD collection that included 2,454 compounds for antifungal activity against the fungal pathogen *Candida auris* identified molecules that inhibit translation initiation (7). Complementary work also identified a compound that potentiates azole activity against azole-resistant *Candida* species through inhibition of efflux (8).

**Fig 1:**
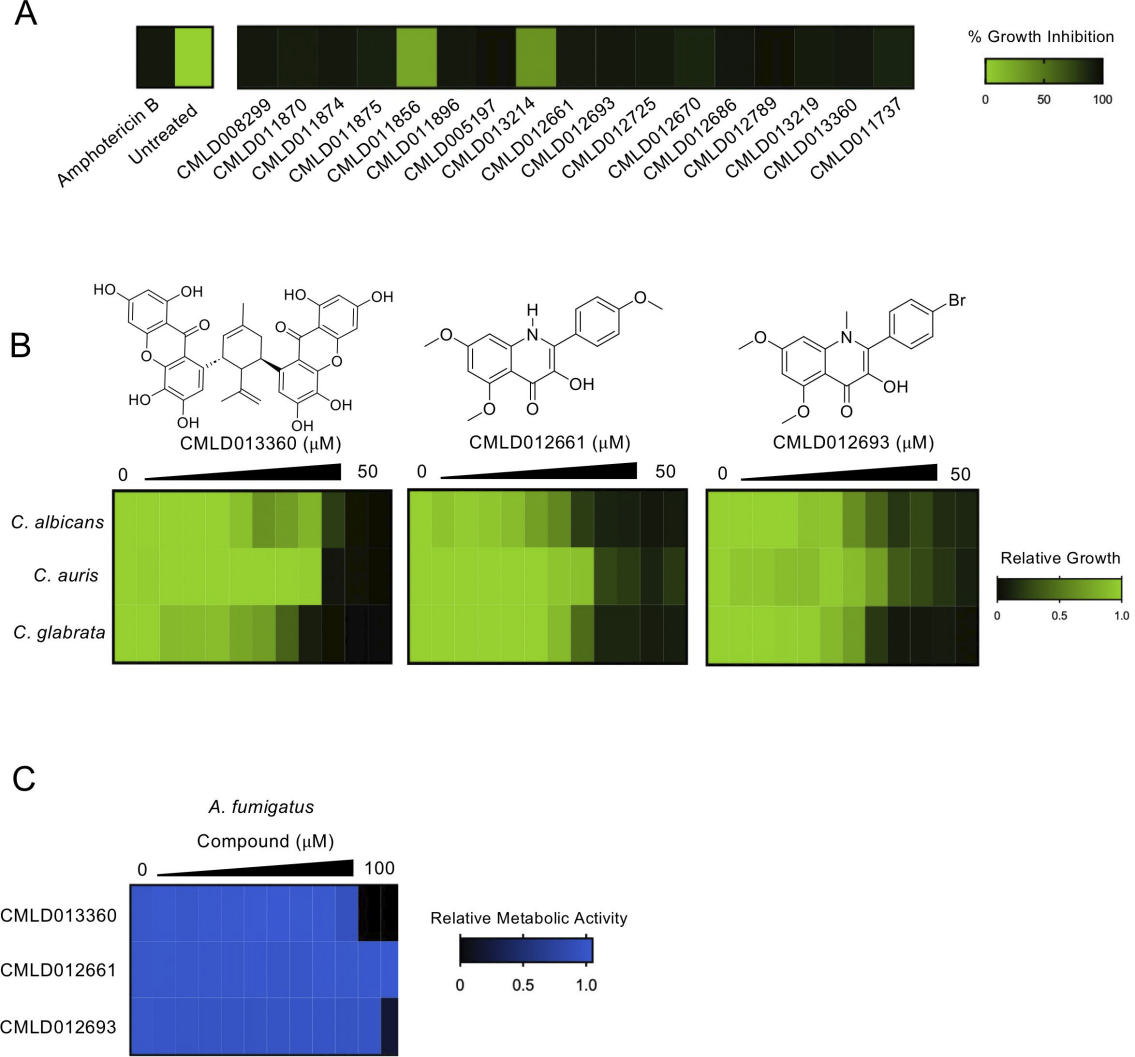
Screening of the BU-CMD chemical library identifies compounds with activity against a clinical isolate of *C. albicans.* (**A**) *C. albicans* (DPL15, a caspofungin-resistant isolate) was grown in RPMI for 48 hours at 30°C with or without compounds (25 µM) from the BU-CMD library. Relative growth was measured by OD_600_. Compounds were considered hits if they reduced growth >80% relative to the median growth observed in the screen. Hit compounds were tested a second time to confirm bioactivity. Growth was normalized and then calculated as a percentage of compound-free controls (see color bar). (**B**) Dose-response assays were performed in a 384-well plate format in technical duplicate, with two-fold dilution gradients of the three prioritized compounds against diverse fungal pathogens. Relative growth was measured by absorbance at 600 nm (OD_600_) after a 48-hour incubation at 30°C in RPMI (see color bar). Data plotted represent results from one of two biological replicates that yielded similar results. (**C**) Dose-response assay was performed as described in (**B**). Standard dye reduction (Alamar blue) assay was used to measure the relative viable *Aspergillus fumigatus* cell number (see color bar). Data plotted represent results from one of two biological replicates that yielded similar results.

To further interrogate the BU-CMD chemical library for antifungal activity, we screened an expanded collection of 3,049 compounds encompassing diverse chemotypes at 25 µM in Roswell Park Memorial Institute (RPMI) 1640 medium against a clinical isolate of *C. albicans*, DPL-15 (9). Cultures were incubated at 30°C for 48 hours before growth was measured by optical density at 600 nm (OD_600_). From this screen, 17 molecules were identified that inhibited growth of *C. albicans* >80% relative to the median growth observed in the screen (Table S1), representing a 0.55% hit rate. To verify results from the primary screen, the compounds were tested again for bioactivity against *C. albicans* using the same conditions as the primary screen, with 15 compounds displaying reproducible activity (Fig. 1A). Of those verified compounds, nine shared a similar scaffold possessing a 3-hydroxyquinolinone, whereas the other six compounds were more chemically diverse (Fig. S1).

In follow-up, we selected three of the most potent molecules to serve as representatives of the distinct chemical scaffolds identified, and used standard dose-response assays to assess their antifungal potency. CMLD013360 is a dimeric xanthone/terpenoid hybrid, generated in the course of synthetic studies toward the griffipavixanthone class of natural products (10). CMLD012661 and CMLD012693 are both 3-hydroxyquinolinones (11), a chemotype known to chelate metal ions (12). In particular, the latter two compounds have some structural similarities to iron chelators including *Pseudomonas* quinolone signal (12) and deferiprone (13). The potency of each compound was evaluated by growing the organisms in microplate format using a medium supplemented with two-fold serial dilutions of each compound as previously described (14). Growth was measured by absorbance at 600 nm (OD_600_) after 48-hour incubation at 30°C in RPMI medium (Fig. 1B). CMLD013360, CMLD012661, and CMLD012693 all displayed activity against *C. albicans* with a minimum inhibitory concentration causing 80% growth reduction (MIC_80_) of 25 µM, 12.5 µM, and 12.5 µM, respectively. Compounds were also active against isolates of the related fungal pathogens *Candida glabrata* (MIC_80_: 12.5 µM, 6.25 µM, and 6.25 µM, respectively) and *C. auris* (MIC_80_:12.5 µM, 12.5 µM, and 25 µM, respectively). Finally, we assessed the potency of the compounds against the evolutionarily divergent mould *Aspergillus fumigatus*. Conidial suspensions (~25,000 conidia/mL) in RPMI medium were added to 96-well plates followed by incubation at 37°C in the presence of compound for 24 hours. Subsequently, a 1:20 dilution of Alamar blue (Invitrogen) in RPMI was added to cells followed by another 24-hour incubation at 37°C (15). Fluorescence was measured (Ex. 560 nm/Em. 590 nm) to quantify metabolic activity. CMLD013360 and CMLD012693 were active against *A. fumigatus* (MIC_80_ 50–100 µM), while CMLD012661 displayed no activity up to 100 µM, the highest concentration tested (Fig. 1B).

Exploiting metal homeostasis has been studied as a strategy to limit fungal infections (16–18). Metals have key roles in all biological systems where they are incorporated into metalloproteins, including enzymes, storage proteins, and transcription factors (19, 20). Common metallonutrients include iron, zinc, copper, calcium, manganese, and magnesium. Iron is essential to the survival of most organisms as an important cofactor for metabolic processes, as well as the transport of oxygen, activation, and decomposition of peroxides, and the reduction of ribonucleotides and dinitrogen (21). Furthermore, in *C. albicans*, iron is required for virulence and pathogenesis (22).

**Fig 2:**
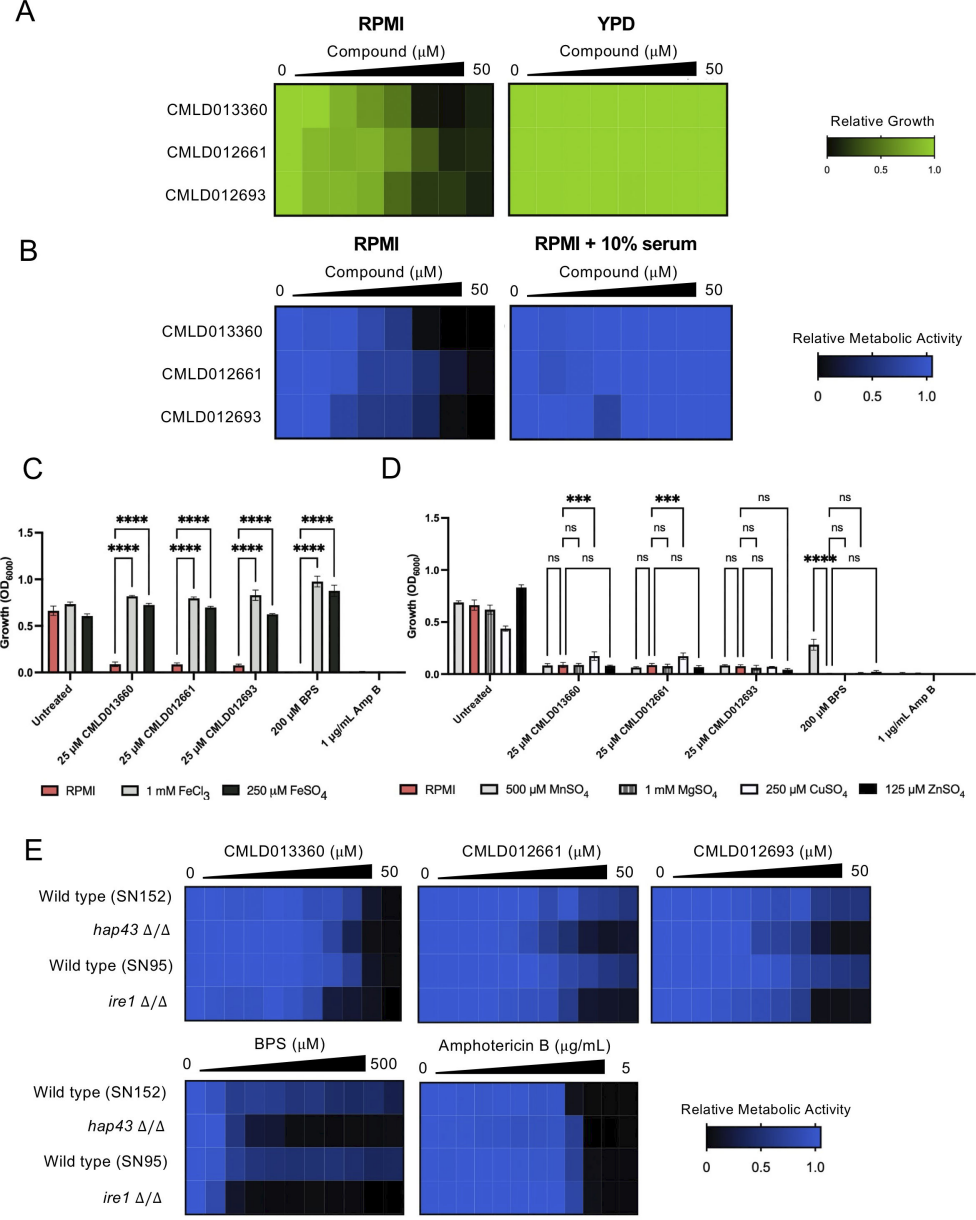
CMLD12661, CMLD012693, and CMLD013360 inhibit *C. albicans* growth through iron chelation. (**A**) Dose-response assays of three prioritized compounds in RPMI and YPD in a 96-well plate format. Relative cell growth was assessed by absorbance at 600 nm (OD_600_) after 48-hour incubation at 30°C (see color bar). (**B**) Dose-response assays of three prioritized compounds in RPMI and RPMI + 10% serum in 96-well plates. After a 48-hour incubation at 30°C, relative metabolic activity was assessed by standard dye reduction (Alamar blue) assay (see color bar). (**C**) Supplementing media with additional iron salt (FeSO_4_ or FeCl_3_) restores fungal growth in the presence of CMLD compounds and the classical metal chelator, BPS (bathophenanthrolinedisulfonic acid). The activity of the known antifungal, amphotericin B (Amp B), was not influenced by iron supplementation. Mutant strains and their respective wild-type parental controls were grown overnight in YPD and then sub-cultured into RPMI supplemented with a concentration of the indicated compound that inhibited growth by 80% in RPMI medium alone. RPMI was also supplemented with 250 µM FeSO_4_ or 1 mM FeCl_3_, as indicated. Relative cell growth was assessed by absorbance at 600 nm (OD_600_) after 48-hour incubation at 30°C. Bars represent the mean of technical triplicates, and error bars represent ±SD (standard deviation) of technical triplicates, ****P ≤ 0.0001. (**D**) Supplementing media with metal salts (MnSO_4_, MgSO_4_, CuSO_4_, and ZnSO_4_) has no to minimal impact on the antifungal activity of compounds. Assay was performed as described in (**C)**. Bars represent the mean of technical triplicates, and error bars represent ±SD of technical triplicates, ****P ≤ 0.0001, and ***P ≤ 0.001. Data plotted represent results from one of two biological replicates that yielded similar results. (**E**) Deletion of *IRE1* or *HAP43* increases the bioactivity of CMLD013360, CMLD012661, and CMLD012693 against *C. albicans*. Parental wild-type strains, and *ire1Δ/ire1Δ* and *hap43Δ/hap43Δ* mutant strains, were grown overnight in YPD and then subjected to dose-response assays in RPMI medium with two-fold dilution gradients of CMLD013360, CMLD012661, and CMLD012693. BPS, a metal chelator, was included as a positive control. Relative cell growth was assessed by a standard dye reduction (Alamar blue) assay after 48-hour incubation at 30°C. For all dose-response assays, growth was averaged between technical duplicates and normalized to drug-free control wells. Heat maps plotted are representative of two biological replicates.

Based on structural insights, CMLD013360, CMLD012661, and CMLD012693 were hypothesized to act as metal chelators (13, 23). We initially evaluated the bioactivity of our three prioritized compounds in RPMI (minimal-metal condition), yeast extract-peptone-dextrose (YPD; nutrient-rich condition), and RPMI supplemented with 10% serum (enriched-metal condition). We observed that all compounds lost bioactivity in YPD and RPMI supplemented with serum as measured by dose-response assays performed using *C. albicans* (Fig. 2A). We hypothesized that in media with increased metal content, the chelation capacity of the compounds might be exceeded and fungal growth restored. Of note, the serum-supplemented condition employed in this assay resulted in extensive *C. albicans* filamentation resulting in unreliable OD_600_ measurements. Thus, the relative viable cell number was quantified by adding a 1:20 dilution of Alamar blue to wells followed by a 3-hour incubation at room temperature. Fluorescence was then measured (Ex. 560 nm/Em. 590 nm) to quantify metabolic activity (Fig. 2B).

To more specifically test the hypothesis that CMLD013360, CMLD012661, and CMLD012693 function as metal chelators, as well as narrow down the metal(s) that might be most relevant to their bioactivity, we treated *C. albicans* with inhibitory concentrations of the molecules of interest in RPMI medium supplemented specifically with an additional iron source (FeSO_4_ or FeCl_3_). The concentration of supplemental iron salt used for these experiments was based on results from dose-response assays defining the highest concentration that did not have a significant inhibitory effect on *C. albicans* growth (Fig. S2). Incubation of *C. albicans* with 25 μM of CMLD013360, CMLD012661, and CMLD012693, as well as 200 μM of the known iron chelator bathophenanthrolinedisulfonic acid (BPS), or 1 μg/mL of the antifungal amphotericin B significantly reduced growth relative to the untreated control (Fig. 2C). Supplementation with 1 mM FeCl_3_ or 250 μM FeSO_4_ restored growth of *C. albicans* in the presence of BPS or the CMLD compounds but had no effect on the antifungal activity of amphotericin B (Fig. 2C). To explore the effect of other metals on compound bioactivity, we repeated the experiment with maximal-tolerated concentrations of MnSO_4_, MgSO_4_, CuSO_4_, and ZnSO_4_. While the addition of CuSO_4_ modestly rescued the growth of *C. albicans* upon incubation with CMLD013360 or CMLD012661, none of the other salts affected the potency of the CMLD compounds (Fig. 2D). Thus, CMLD013360, CMLD012661, and CMLD012693 likely exert antifungal activity by chelating iron.

Finally, to support the hypothesis that our prioritized compounds act through iron chelation, we performed dose-response assays with *C. albicans ire1Δ*/*ire1Δ* and *hap43Δ*/*hap43Δ* mutants. *IRE1* encodes a protein kinase that has an essential role in iron uptake and *C. albicans* virulence, whereas *HAP43* encodes a transcriptional regulator, which under low-iron conditions represses the expression of genes encoding iron-dependent proteins (24, 25). Given the important role of these genes in iron homeostasis, we predicted that deletion of these genes would confer hypersensitivity to compounds that chelate iron. Similar to what we observed with BPS, culture of the *ire1Δ*/*ire1Δ* or *hap43Δ*/*hap43Δ* strains with CMLD012661 and CMLD012693 resulted in eight-fold hypersensitivity to the compounds compared to their respective wild-type control strains (Fig. 2E). However, deletion of *IRE1* or *HAP43* resulted in only approximately two-fold hypersensitivity to CMLD013360 relative to wild type, suggesting this dimeric xanthone may affect iron homeostasis in a manner distinct from that of the other two compounds or may have additional non-chelation-related antifungal activities. Overall, results support the conclusion that CMLD013360, CMLD012661, and CMLD012693 exert their antifungal activity primarily through iron chelation.

In summary, this work characterizes the activity of iron chelators against important fungal pathogens. Future studies will be useful in further refining our understanding of the growth inhibitory effects of antifungal metal chelators. These insights gained could facilitate additional efforts toward the development of urgently needed new therapies to combat fungal infections.

## SUPPLEMENTAL MATERIAL

Fig S1 and Fig S2 spectrum.04095-23-s0001.pdf Supplemental figures with legends.

Table S1 spectrum.04095-23-s0002.xlsx Chemical screen results.

ASM does not own the copyrights to Supplemental Material that may be linked to, or accessed through, an article. The authors have granted ASM a non-exclusive, world-wide license to publish the Supplemental Material files. Please contact the corresponding author directly for reuse.
