# Supplementary material for: A chemical screen identifies structurally diverse metal chelators with activity against the fungal pathogen Candida albicans
Source: Microbiol Spectr. 2024 Feb 20;12(4):e04095-23. doi: 10.1128/spectrum.04095-23 (PMC10986608; doi:10.1128/spectrum.04095-23)
Supplement: Fig S1 and Fig S2 — Supplemental figures with legends. [file spectrum.04095-23-s0001.pdf]

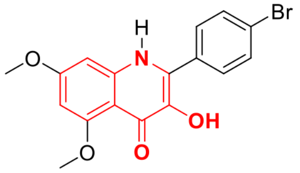

CMLD011870

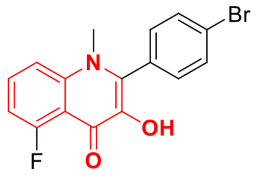

CMLD012686

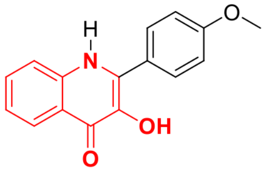

CMLD012725

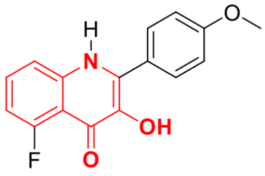

CMLD011874

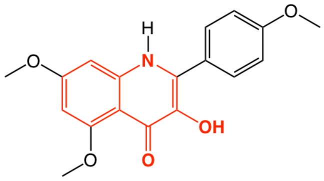

CMLD012661

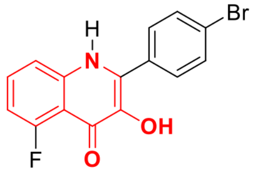

CMLD011875

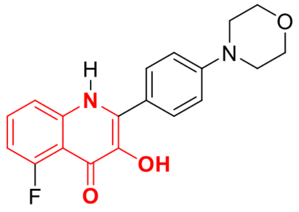

CMLD012670

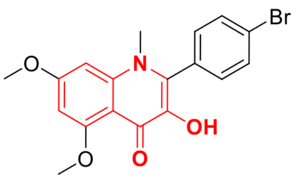

CMLD012693

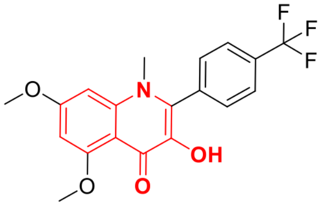

CMLD011896

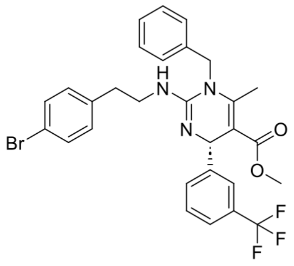

CMLD005197

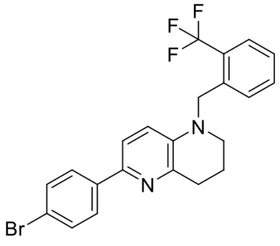

CMLD008299

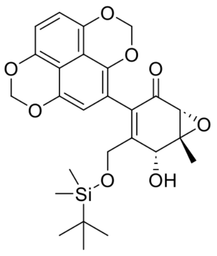

CMLD012789

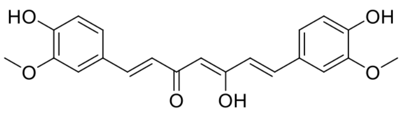

CMLD011737

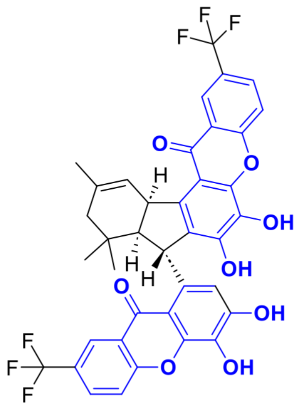

CMLD013219

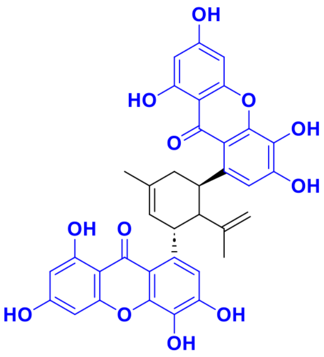

CMLD013360

Supplemental Figure 1. Chemical structures of BU-CMD hit compounds. Red colour depicts compounds with a 3- hydroxyquinolinone scaffold. Blue colour depicts compounds with a dimeric xanthone scaffold.

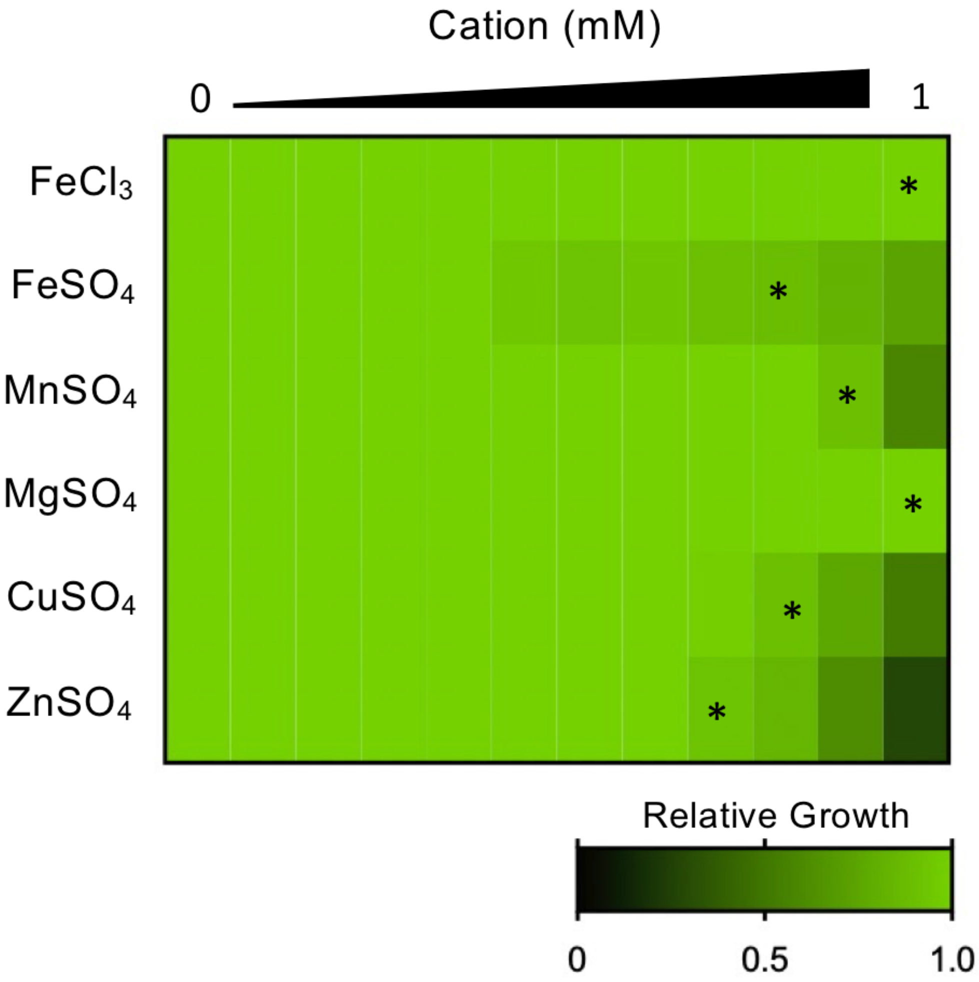

Supplemental Figure 2. Dose response assays to determine the highest concentration of metal salt that does not significantly inhibit the growth of *C. albicans*. Dose-response assays with two-fold dilution gradients starting at 1 mM of $FeCl_3$, $FeSO_4$, $MnSO_4$, $MgSO_4$, $CuSO_4$, or $ZnSO_4$, in RPMI. Relative *C. albicans* growth was measured by absorbance at 600 nm (OD_{600}) after 48 hours at 30 °C. The maximal concentration of supplemental metal salt that did not have a significant inhibitory effect on growth (< 20% inhibition, indicated by asterisk) was chosen for subsequent media supplementation assays. Data plotted represent results from one of two biological replicates that yielded similar results.
